# Use of hydrogen deuterium exchange mass spectrometry in tandem with modern structural biology

**DOI:** 10.1042/BCJ20250131

**Published:** 2026-04-13

**Authors:** Hunter G. Nyvall, Alexandria L. Shaw, Emma E. Walsh, Hirsh Bhatti, John E. Burke

**Affiliations:** 1 Department of Biochemistry and Molecular Biology, The University of British Columbia, Vancouver, British Columbia, V6T 1Z3, Canada; 2 Department of Biochemistry and Microbiology, University of Victoria, Victoria, British Columbia, V8W 2Y2, Canada; 3 University of Victoria Genome BC Proteomics Centre, Victoria, BC, Canada

**Keywords:** allostery, AlphaFold3, Cryo EM, HDX-MS, hydrogen exchange, protein dynamics

## Abstract

Hydrogen–deuterium exchange mass spectrometry (HDX-MS) is an established technique that measures the exchange rate of amide hydrogens, with this exchange rate being a surrogate for protein conformational dynamics. Advances in instrumentation, automation, and data analysis have transformed HDX-MS into a high-throughput and highly reproducible method capable of probing complex biological systems and addressing key questions that have been challenging to study by other structural biology approaches. By enabling measurement of amide exchange rates and mapping of differential exchange between distinct conformational states, HDX-MS provides insight into both allosteric transitions and protein interaction interfaces. Recent advances in the capabilities of artificial intelligence (AI) have been rapidly adopted by structural biology, leading to an unprecedented expansion in the quantity and accessibility of structural predictions, underscoring the need for experimental methods to validate these predicted models and provide insight into both epitopes and allosteric conformational changes. This is particularly critical for non-evolutionarily driven interactions such as antibodies, nanobodies, and artificially designed proteins, where AI technologies can yield false positives. This review highlights how HDX-MS can be integrated synergistically into modern structural biology workflows (cryo-EM, X-ray crystallography, and AI-enabled modeling) and how combining these approaches can be powerful to advance our mechanistic understanding of complex biological processes.

## Introduction

Hydrogen–deuterium exchange mass spectrometry (HDX-MS) is a well-established technique for probing conformational dynamics and has become an indispensable tool for biophysical analysis of protein complexes. Its ability to obtain peptide-level resolution enables structural insights into proteins and protein systems that have been notoriously difficult to characterize by other structural biology approaches. Recent advancements in mass spectrometry, chromatography, and automation have improved HDX-MS throughput and reproducibility, making it more accessible in both academic and industry settings [1–6]. As a result, HDX-MS supports a wide range of applications and has become a standardized method for mapping protein–protein interactions (PPIs) and protein-ligand binding sites in a relatively rapid and cost-effective manner, with this approach being widely adopted for antibody epitope mapping in biopharmaceutical research.

The most transformative shift in structural biology in decades has been driven by the widespread adoption of artificial intelligence (AI)-enabled deep learning models for predicting protein structure and interactions, and *de novo* design of proteins. This includes isolated protein structure prediction algorithms such as AlphaFold2/3 and RoseTTAFold [7–9]; models for protein–protein interfaces (AlphaFold3, AlphaFold Multimer) [7,10], predictors of protein-small molecule binding pockets (AlphaFold3, Boltz, Chai) [7,11,12]; and *de novo* design of targeted binding partners, including peptides, small molecules, and proteins [13–24]. While these technologies have foundationally reshaped structural biology, a persistent challenge is the generation of false positives, particularly for non-evolutionarily driven interactions. These still require rapid validation in a biologically relevant context. High-throughput screening techniques such as protein arrays and phage display [25,26] can identify binders, with kinetic assays including bio-layer interferometry [27] and surface plasmon resonance [28] assessing binding kinetics. However, these provide no structural information on interaction surfaces. Although X-ray crystallography and cryo-electron microscopy (cryo-EM) offer high-resolution analysis of epitopes, they are relatively low-throughput techniques, making them poorly suited to validate AI-generated predictions at scale. NMR (nuclear magnetic resonance) is also a powerful structural biology technique, though it is usually limited to protein complexes <100 kDa, and therefore is not as applicable for analysis of large protein complexes. For this reason, our focus will be on discussing HDX-MS use in tandem with cryo-EM, X-ray crystallography, and AI-enabled modeling. HDX-MS offers a solution to gain insight into protein dynamics that are not achievable using X-ray or cryo-EM and allows for testing of AI-predicted interactions. The throughput of HDX-MS experiments, where testing of a protein–protein binding site can be completed in a single day, compared with the more extensive timelines of cryo-EM and X-ray crystallography, allows for data to be produced at a scale compatible with rapid validation. The need for such capabilities is essential in modern structural biology workflows.

It is important to highlight the differences between structural biology techniques and HDX-MS. X-ray crystallography and cryo-EM allow for analysis of the electron density/potential of ordered regions of proteins, with cryo-EM also allowing for analysis of multiple conformations. By modeling amino acids into the electron density present in X-ray and cryo-EM data, it is possible to provide direct information on the three-dimensional arrangement of amino acids within protein complexes. Any regions that are disordered and can adopt multiple conformations lack electron density, with no information being revealed using these techniques. In contrast, HDX-MS reports on the dynamics of the protein backbone, providing information for both ordered and disordered regions of proteins; however, no information is provided on the three-dimensional arrangement of the atoms. In this way, HDX-MS and other structural techniques provide distinct and orthogonal information that synergistically provide a more complete snapshot into protein structure/function. Intriguingly, there are multiple examples of proteins with very similar protein folds that show dramatic differences in their H/D exchange rates [29], highlighting the differences between protein structure and protein dynamics.

This review will outline the theoretical framework, experimental considerations, and broad application of HDX-MS that underscore its growing integration into modern structural biology. This review is aimed at structural biologists and biochemists interested in leveraging HDX-MS alongside complementary structural biology approaches (cryo-EM, X-ray crystallography, NMR, and AI-enabled modeling) to enhance mechanistic insight into their biological system of choice. We will focus on published examples of how HDX-MS allows for the rapid identification of protein–protein interfaces, how it can guide assessment and validation of AI-predicted protein–protein interfaces, and how it has provided insight into the molecular basis of disease-linked mutations. Examples of how HDX-MS revealed important insight into the assembly of complexes analyzed by X-ray crystallography and cryo-EM will also be highlighted. Altogether, this review aims to summarize how HDX-MS is poised to have an important role as a complementary tool to modern structural biology, particularly in its ability to probe not only protein structure but also protein dynamics.

## Practical considerations in HDX-MS experiments

Hydrogen–deuterium exchange (HDX) experiments utilize the exchange of backbone amide hydrogens with solvent [30,31], with contemporary approaches predominantly using mass spectrometry (MS) to quantify deuterium incorporation [32,33]. Exchange rates at individual amide hydrogens are dependent on the extent of their involvement in secondary structure hydrogen bonding (i.e. stability of protein secondary structure) and, to a lesser extent, their solvent accessibility [34,35]. Additional factors, including pH, temperature, and the influence of surrounding amino acids, also modulate exchange but can be controlled for experimentally [36,37]. Due to space limitations, we will not exhaustively summarize the extensive literature on the mechanistic basis for how amide hydrogens exchange with solvent, or the wide diversity of how H/D exchange can be utilized. Readers are advised to consult recent comprehensive reviews on the topic for further details [2,3].

**Figure 1:**
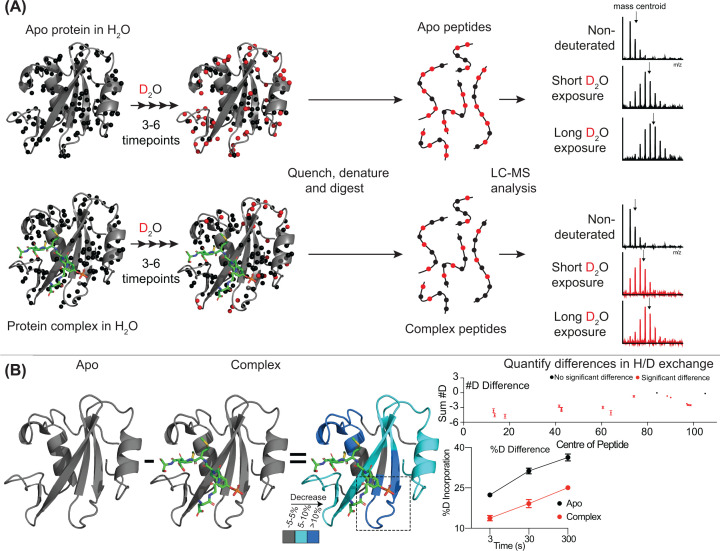
Summary of HDX-MS experimental methodology (**A**) Schematic representation of the typical workflow of a differential HDX-MS experiment, from deuterium labeling to mass spectrometry analysis. Black spheres represent the exchangeable amide hydrogens; red spheres show an approximate representation of amide hydrogens that have been replaced with deuterium to visualize labeling. Mass spectra from differing protein states and labeling timepoints show how deuterium incorporation affects readout. Mass centroids of each spectrum are denoted with an arrow, showing how this can be used to identify differences in deuterium uptake. (**B**) Example of a differential HDX-MS experiment to determine the location and extent of changes in deuterium uptake upon ligand binding. %D difference graph shows the exchange profile in the unbound and ligand-bound conditions for the peptide located within the dotted box of the structure. #D graph shows the sum of the #D difference over the entire time course. Each point represents the central residue of a single peptide, with significant differences in exchange shown in red. HDX-MS data based on the cSH2 of PIK3R1 binding to a phosphorylated peptide [44].

This section focuses on the most common application of HDX-MS: differential HDX experiments that compare H/D exchange rates between unique protein states (Figure 1). These experiments are most straightforward when the conformational states of interest can be stabilized, as highly dynamic systems are more challenging because HDX-MS reports on conformational ensembles. These systems can still be investigated, particularly if binding partners uniquely enrich distinct conformational states; however, they generally require a greater familiarity with the protein system under study. Any differential HDX-MS experiment can be broken down into four fundamental steps: (i) Design of the deuterium exchange reaction, (ii) optimization of quench, digestion, and peptide separation, (iii) MS analysis of deuterated peptides, and (iv) data analysis of H/D exchange.

Critical to any HDX-MS experiment is the careful design of the deuterium exchange reaction, including the protein and binding partner concentrations, buffer composition, D_2_O content, and the range of labeling timepoints. In a typical HDX-MS workflow, samples are exposed to a deuterated solvent for defined time periods. Backbone amides span a wide range of intrinsic exchange rates, with unstructured regions exchanging on the order of milliseconds to seconds, while highly structured, stable regions can take days to fully exchange [38–40]. Because of this, the time scale of deuterium labeling should be carefully selected to make sure that a broad enough spectrum of amide exchange rates is interrogated. Experiments commonly span, at a minimum, four orders of magnitude in time, with the short incubations of 1–10 s and longer timepoints lasting hours. Assessing the shortest exchange time periods can be complicated by fully automated modern robotic systems that cannot access extremely short time points of exchange. This results in these time points being frequently undersampled in fully automated HDX approaches. This can be addressed using manipulation of buffer pH, but careful analysis that protein conformation is stable over various pHs is essential [40]. As the H/D exchange reaction is incredibly pH and temperature-sensitive, buffer composition must be identical across all comparative states, and all samples must be treated in the same way. This is to ensure that any exchange differences are not a result of either of these conditions being altered over the course of the experiment. Protein quantity should be identical across samples and optimized to obtain adequate signal intensity (usually 5–50 pmol per sample), although this can vary depending on the sensitivity of the mass spectrometer used. The percentage of D_2_O used during the labeling can be tuned to increase or decrease the mass shift as needed.

Frequently, the most important step in a differential HDX-MS experiment is the correct setup of the protein and ligand concentrations used. Ideally, concentrations should be chosen to maximize binding occupancy and maintain protein stability and solubility throughout deuterium labeling [41]. Low affinity interactions may require additional optimization of sample volumes and buffer composition [42]. This is important because the resulting mass spectra represent a summation of the deuteration profiles of each molecule in the sample. If unwanted protein states are present, this data will be indistinguishable from the target protein state for analysis [43].

Upon completion of labeling, the exchange reaction is halted by simultaneously lowering the temperature to 0°C and reducing the pH to ∼2.5, the global minimum of the acid-base-catalyzed exchange reaction [45,46]. This quenching step accomplishes two objectives: it initiates denaturation and reduction of the protein, and minimizes further deuterium incorporation [46,47]. A denaturant (e.g. guanidine hydrochloride) and reducing agent (TCEP (tris(2-carboxyethyl)phosphine)) [48,49] are typically included to fully unfold and reduce the protein, helping to facilitate downstream proteolysis. After quenching, samples are digested by an acid-functional protease (e.g. pepsin, nepenthesin, or fungal proteases). Digestions can be performed either in solution or in an online fluidics system using immobilized protease columns. This online configuration allows for digestion to be immediately followed by peptide separation by liquid chromatography and elution directly onto the mass spectrometer. To ensure high-quality data and reduce back exchange, the total amount of time between quenching and mass analysis should be minimized, and at an absolute minimum, it needs to be standardized for all samples. All steps need to be carried out at low temperatures and a pH of ∼2.5 to limit back exchange during these steps, although there can be advantages of slightly increased temperatures during protein digestion to increase proteolysis efficiency.

When analyzing the obtained mass spectra, the isotopic distributions corresponding to each peptide are compared between states to determine any changes in mass, allowing for the identification of regions with differences in deuterium incorporation (Figure 1B) [50]. During data processing, spectra should be checked to confirm the accuracy of peptide identification and calculated levels of deuterium incorporation, either manually or with dedicated HDX-MS software packages to ensure assignment accuracy [51]. This step becomes more important as larger protein systems are studied, as incorrect peptide identifications become more likely as the possible number of peptides increases. Clear analysis criteria to prevent false positives is essential. Recent advances in the use of data-independent acquisition have greatly increased the ability to automate HDX data analysis and reduce peptide assignment errors by using information present in the fragment spectrum [51].

While minimizing and standardizing the back exchange that occurs during analysis is important, it can also be useful to measure the absolute back exchange for any protein system [52]. The best practice is to include a maximally deuterated control to estimate back-exchange levels on a per-peptide basis. However, this can frequently be challenging in complicated protein systems where it is impossible to achieve a maximally deuterated sample without significant aggregation. At a minimum, differential HDX experiments should be corrected for the D_2_O content of the labeling buffer. If relative exchange values are used that do not account for back exchange, it is essential that there is a benchmark system in place that establishes the amount of back exchange expected in the fluidics system. Peptides exhibiting high sample-to-sample carryover should also be checked for when reviewing raw data, as they can mistakenly be mixed up with EX1 kinetics (see more details below). Carryover should be monitored by collecting data from system blanks and attempting to identify troublesome peptides, with optimized buffers for cleaning of the proteolysis and trap system being useful in preventing carryover [43,53].

A major consideration during data processing is the kinetic regime of amide exchange. Amides fluctuate between open and closed states, with only the open state accessible to exchange [30]. In most cases, the open–closed transition is faster than the deuterium exchange reaction, resulting in a progressive shift in m/z values over time, known as EX2 kinetics. Less frequently, the open–closed transition is slower than exchange, resulting in EX1 kinetics. Here, peptides populate either fully exchanged or non-exchanged states, producing bimodal isotopic distributions whose relative intensities change over time [2]. True EX1 behavior is uncommon and requires careful validation, as pseudo EX1 kinetics can be observed due to a variety of factors (partial binding occupancy, mixed population heterogeneous samples, and sample carryover). Careful inspection of isotopic envelopes can identify the presence of EX1 kinetics; however, full validation of true EX1 kinetics is non-trivial and requires extensive controls on the protein preparation and fluidics to prove that the EX1 kinetics observed are due to dynamics within a homogenous protein sample. When EX1 kinetics are present, raw spectra and deuterium-incorporation plots showing both populations should be provided [3]. For a more comprehensive overview of experimental considerations of HDX-MS, please consult the following reviews [2–4,54,55]. Differences in HDX can be visualized using butterfly plots, Woods plots, or peptide-level deuterium incorporation plots. For all published HDX-MS studies, the data underlying all deuterium incorporation for every peptide at every time point, as well as the standard deviation of this measurement, should be included.

In differential HDX-MS experiments, protections from exchange at early timepoints (e.g., 3 s) typically reflect a transition from a disordered to an ordered state—often indicative of ligand-induced stabilization of flexible regions. Conversely, protections that appear only at late timepoints (e.g., 3000 s) are characteristic of highly stable, structured regions being protected. Fundamentally, differences that occur at different time points provide no information on whether changes are driven through direct interactions or through allosteric effects. This makes a definitive interpretation of a protein or small molecule binding surface by HDX-MS challenging and is frequently one of the most misinterpreted aspects of HDX data analysis. In this way, HDX-MS serves as a kind of “molecular ruler,” enabling detailed interrogation of alterations in protein dynamics. True validation of differences in protein dynamics caused by either direct or allosteric effects requires further validation using a variety of other biophysical approaches and cannot be analyzed simply by examining the deuteration curves. We will discuss in the PPI section how AI-enabled modeling can help to partially address this concern.

## Protein–protein interactions

PPIs define myriad biological functions and are essential in cellular signaling pathways. PPIs in cell biology span an enormous dynamic range, from fleeting millisecond encounters that enable rapid signaling to long-lived highly stable assemblies. There are many modulators of protein–protein interfaces, including membranes, scaffolds, small molecules, and post-translational modifications. Understanding how proteins interact with binding partners and how these interactions are modulated is essential in elucidating protein function. HDX-MS is particularly well suited to address numerous aspects of PPIs, including analysis of protein interfaces formed at intrinsically disordered regions (IDRs) [56], protein complexes that form on membranes [57,58], allosteric conformational changes driven by complex formation [59,60], and epitopes for biotherapeutics [61–63].

HDX-MS has become an increasingly common method to complement the high-resolution structural data obtained from X-ray crystallography and cryo-EM in the study of PPIs. Advances in cryo-EM, including software developments that improve data processing [64,65] and the use of direct electron detectors that improve data quality and, in turn, resolution [66], have enabled investigations of large dynamic protein systems, with atomic-level resolution achieved in some cases [67,68]. However, these techniques often provide static, discrete snapshots of protein conformations, especially in the case of X-ray crystallography, and often fail to capture the dynamic behavior that is inherent to proteins [69]. HDX-MS excels in this context, highlighting the clear advantage of combining it with other structural techniques for comprehensive structural characterization. While cryo-EM and X-ray crystallography excel at defining the exact molecular basis for how proteins interact, there can be severe challenges in the generation of this data, with the speed of data acquisition being several orders of magnitude slower than structural prediction. In contrast, HDX-MS provides lower-resolution structural insights in a more streamlined and accessible manner, allowing the feasibility of a protein target to be determined before committing to high-resolution structural studies. The following examples illustrate how HDX-MS has been employed to interpret and complement results obtained from these high-resolution techniques.

The following section outlines specific examples of how HDX-MS has been applied to study all these different aspects of protein–protein interfaces, focusing on analysis of the phosphoinositide kinases.

### HDX-MS analysis of protein interactions of the phosphatidylinositol 4-kinases (complement to cryo-EM and X-ray crystallography)

Phosphatidylinositol 4-kinases are lipid kinases that generate phosphatidylinositol 4-phosphate at multiple cellular membranes, with there being four distinct enzymes in humans [70,71]. We will describe the use of HDX-MS to examine the protein interactions of the PI4KA and PI4KB enzymes, with these being a useful model as their activity is regulated by a plethora of lipid and protein binding partners.

The PI4KB enzyme is primarily localized at the Golgi and trans-Golgi Network (TGN), with multiple protein binding partners controlling its localization and activity, including the GTPase Rab11, the Golgi resident protein acyl-CoA binding protein 3 (ACBD3), and the TGN localized protein armadillo-like helical domain containing 3 (ARMH3). PI4KB acts as a useful case study for exploring how HDX-MS can nicely complement other structural biology approaches. This is for three primary reasons: (i) X-ray analysis of PI4KB binding to Rab11 could not unambiguously define what the true biological interface was due to crystal packing artifacts, (ii) PI4KB binds to ACBD3 and ARMH3 through an IDR that becomes ordered upon complex formation, and (iii) the interactions of PI4KB with some of its protein partners are structurally uncharacterized and can be used to test AI-based predictions of PPIs.

**Figure 2:**
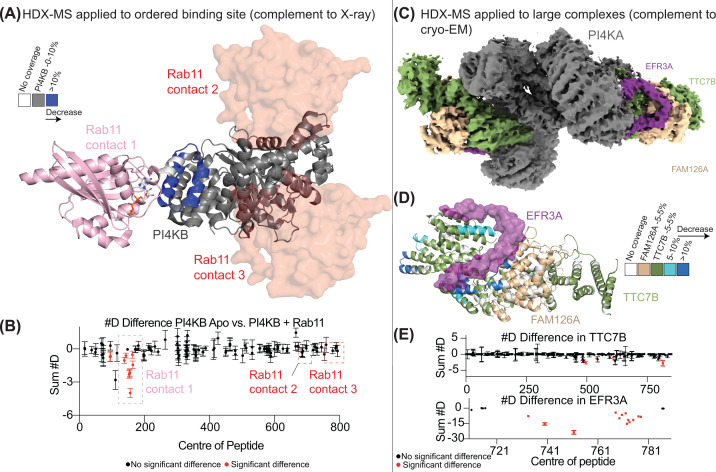
HDX-MS provides a powerful complement to established structural biology techniques like X-ray crystallography and cryo-electron microscopy (**A**) Structure of PI4KB binding to Rab11, with putative binding surfaces indicated. HDX-MS was used to identify the true PI4KB–Rab11 interface, as the crystal structure had three unique interfaces due to crystal packing artifacts (PDB 4D0L) [73]. The blue regions in PI4KB indicate HDX protections upon Rab11 binding. (**B**) #D difference graph showing a decrease in deuterium exchange in PI4KB upon Rab11 binding [73,74]. (**C**) Cryo-EM map of the PI4KA complex bound to EFR3A, showing that EFR3A interacts with TTC7B and FAM126A (EMD-44413) [76]. (**D**) Structure of TTC7B and FAM126A in complex with EFR3 with decreases in deuterium exchange upon EFR3A binding mapped, validating the interaction is PI4KA-independent (PDB: 9BAX) [76]. (**E**) #D difference graphs of TTC7B and EFR3A upon binding EFR3A or the TTC7B-FAM126A complex, respectively. This figure contains data from [73,74,76].

PI4KB binds to the GTPase Rab11, with this interaction not being critical in controlling PI4KB localization, where PI4KB plays a non-catalytic role in regulating a pool of Rab11 instead [72]. The X-ray crystallography structure of PI4KB bound to Rab11 showed three possible interfaces for Rab11 bound to PI4KB (Figure 2A), with in-solution analysis showing a 1:1 binding stoichiometry [73], signifying that two of these interfaces were crystallographic artifacts. HDX-MS analysis showed that Rab11 caused significant decreases in exchange only at the ordered helical domain interface, with no differences at either of the two other putative interfaces (Figure 2B) [73,74]. Intriguingly, protein interface predictions suggested one of the other sites as the most likely interface [7,75]. This interface is partially composed of the GTP-bound nucleotide of Rab11, which likely is what caused inaccuracy in the AI prediction of the protein–protein interface, as it heavily weights the co-evolution of binding interface residues. Overall, this highlights the critical role of HDX-MS in modeling the correct PI4KB–Rab11 interface.

HDX-MS analysis is particularly useful in determining disorder-to-order transitions of IDRs upon formation of complexes with their binding partners [77–79]. This is due to the formation of secondary structure upon protein engagement, leading to large decreases in H/D exchange primarily at the short deuterium exposure time points. PI4KB engages with its Golgi binding partner ACBD3 through the engagement of a disordered N-terminus of PI4KB with the Q domain of ACBD3 [80]. This data corresponded nicely to NMR analysis that showed formation of a single helix in PI4KB, forming a coiled coil with the Q domain [81].

HDX-MS has also been useful in the analysis of protein binding partners of the large multiprotein PI4KA complex. PI4KA is primarily located at the plasma membrane, and forms a trimeric complex with its regulatory protein TTC7 and FAM126 [82,83]. The main driver localizing PI4KA to the plasma membrane is binding to the lipidated protein EFR3. HDX-MS showed that the flexible C-terminus of EFR3 binds to an extensive interface on the TTC7 and FAM126 regulatory proteins and confirmed that FAM126A and TTC7B alone were sufficient for EFR3 binding. This was consistent with the cryo-EM model suggesting the interaction is mediated solely through these proteins (Figure 2C–E). Additionally, the PI4KA complex can be regulated by the protein phosphatase calcineurin [84]. Computational methods originally identified a possible calcineurin binding motif in the disordered C-terminal tail of the FAM126A regulatory protein. HDX-MS analysis validated this interface, but unexpectedly identified a secondary calcineurin binding site in two disordered loops spanning the horn and dimerization domains of PI4KA [84,85]. The interaction with calcineurin was at the limits of protein interactions that HDX-MS can measure, as this was a very transient, low-affinity interface. This required careful tailoring of the experimental setup, where protein incubation was carried out in low volume at high protein concentrations using short deuterium exposure, low temperature, and low pH to capture differences in the rapidly exchanging amides.

### HDX-MS analysis of protein interactions of the phosphatidylinositol 4-kinases (complement to AI-enabled modeling)

In recent years, there has been an explosion of tools that allow for AI-enabled molecular modeling of protein–protein interfaces. Structural MS is taking an important role in the validation of these studies, including the use of chemical cross-linking and HDX-MS. This has led to several studies that have combined AI-enabled modeling and HDX-MS to either validate or disprove AI-predicted interfaces [62,86–96]. This includes studies that have built HDX-MS restraints into LLM (large language model) pipelines for prediction of protein interaction surfaces [62,92], with most others focused on correlating HDX-MS data with models generated by AlphaFold3, AlphaFold-multimer, AlphaFold2, or RoseTTAfold.

Analysis of the results generated by the combined analysis of HDX-MS and AI-enabled modeling is not always straightforward, as there can be challenges in complexes that undergo large conformational changes upon formation. This is a well-established issue already for HDX-MS analysis, as many times HDX differences at the protein binding interface can be accompanied by allosteric changes at distant regions. While AI modeling can partially answer this issue as it can make a prediction of the interface, it can give varying levels of confidence, particularly for weak or transient interactions that may have a certain degree of plasticity.

**Figure 3:**
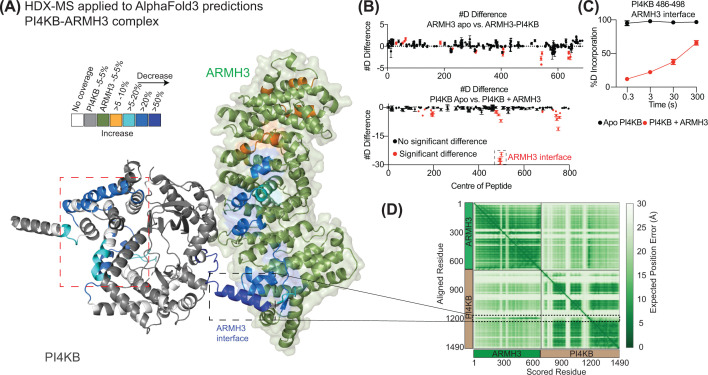
HDX-MS as a complement to AlphaFold3 AI-enabled protein predictions (**A**) AlphaFold3 model of PI4KB binding ARMH3, with PI4KB shown as a cartoon, and ARMH3 shown as a cartoon with transparent surface. Differences in HDX-MS are colored according to the legend. The PI4KB ARMH3 interface is highlighted, with decreases in the helical/kinase domain, shown in the dotted red box, which could be either allosteric conformational changes or incorrectly predicted data [97]. (**B**) #D difference graph showing differences in deuterium exchange in both PI4KB and ARMH3 upon complex formation [97]. (**C**) Data from a single peptide spanning the ARMH3 interface in PI4KB highlight a disorder-to-order transition in this region upon complex formation [97]. (**D**) Predicted aligned error from AlphaFold3 modeling, with the ARMH3 interface in PI4KB highlighted.

A key example of this is the interaction of PI4KB with the protein ARMH3. HDX-MS analysis of both ARMH3 and PI4KB upon complex formation identified multiple regions with exchange differences. The primary binding interface on PI4KB for ARMH3 is in a disordered loop in the kinase domain, annotated as the ARMH3 interface in Figure 3A–C [97]. Intriguingly, analysis of the AlphaFold3 predictions of this interface shows a low predicted aligned error (pae, a measurement of AlphaFold accuracy) for this loop (Figure 3D), but the pae values for the rest of PI4KB are much higher, suggesting that the prediction of the rest of PI4KB relative to ARMH3 may be incorrect. This is supported by the remaining differences in exchange in PI4KB upon ARMH3 binding being observed in the helical and kinase domains, distant from the predicted interface. However, it is important to highlight that this prediction may be correct and that these changes could be driven by allosteric effects. Even with ambiguity on the full molecular details of the interface, this still provides valuable biological insight into the complex under study. Mutation of a single residue in the ARMH3 binding interface identified by HDX-MS and predicted by AlphaFold3 completely blocked complex assembly [97].

These caveats should be considered when HDX-MS data are used in tandem with AI-enabled modeling, particularly when it is used as a tool to validate interfaces. It is critical to understand that proper interpretation of results can be complicated by the fact that allosteric HDX-MS differences may occur upon protein complex formation.

### HDX-MS analysis of protein interactions of the phosphoinositide 3-kinases (PI3Ks) (analysis of allosteric conformational changes upon protein/membrane binding)

In many cases HDX-MS is very powerful in observing large allosteric conformational changes that occur when signaling enzymes bind to their upstream activators. This is particularly true for class I phosphoinositide 3-kinases. The class I PI3Ks are lipid kinases that act as master regulators of cellular growth, with them having myriad protein binding partners that both recruit and activate them on the plasma membrane [98]. They are large (∼200 kDa) heterodimeric complexes composed of a p110 catalytic subunit and a regulatory subunit. They act as a key example for how HDX-MS can be used to study large allosteric conformational changes upon protein binding, as well as membrane-localized PPIs.

**Figure 4:**
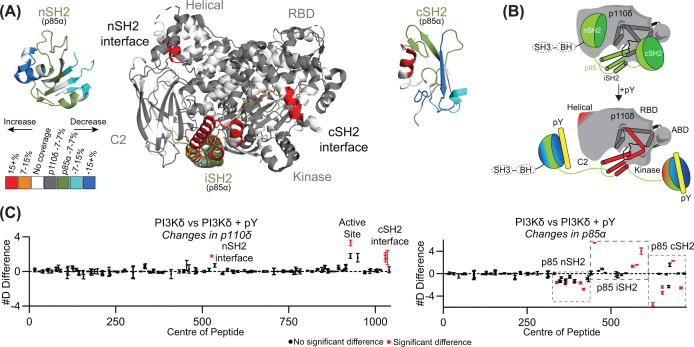
HDX-MS reveals allosteric changes upon phosphopeptide binding in class IA PI3Ks (**A**) Upon pY binding to p85α’s nSH2 and cSH2 domains, a decrease in deuterium exchange was observed. In contrast, increased deuterium exchange occurred within p110δ. Without the structure of the p110δ–p85α complex, these changes could be differently interpreted. (PDB: 5DXU) [107]. Figure adapted from [101]. (**B**) Cartoon schematic of changes described in panel (A). Binding of phosphopeptide to the nSH2 and cSH2 domains of p85α breaks the inhibitory interactions between the p110δ and p85α regulatory subunits. Figure adapted from [101]. (**C**) #D graphs show the sum of the #D difference for p110δ and p85α, showing increases in p110δ and both decreases and increases in p85α. The domains described in panel (A) are highlighted. This figure contains data from [101].

The class IA PI3Ks are all activated through engagement of their p85 regulatory subunits with bis-phosphorylated pYXXM motifs (referred to as pY for the rest of the manuscript) present in receptor tyrosine kinases and their adaptor proteins. HDX-MS was used to study all of the class IA catalytic subunits and how pY engagement alters the dynamics of both the catalytic and regulatory subunits [44,99–104]. A representative example of pY activation is the PI3Kδ complex (composed of a p110δ catalytic subunit and a p85α regulatory subunit) (Figure 4) [101]. The engagement of pY with the nSH2 and cSH2 is observable as decreased exchange throughout most of these domains; however, there are extensive regions in the catalytic subunit showing increased exchange. This was interpreted as a disruption of the inhibitory interfaces between the catalytic and regulatory subunits, with domain deletion HDX-MS experiments validating this approach [104]. However, it is absolutely critical to define that interpreting these experiments would have been extremely difficult in the absence of structural models of the inhibited PI3K states [105,106], once again highlighting the complementary nature of HDX-MS in modern structural biology.

PI3Ks are recruited to the plasma membrane by a variety of membrane-localized proteins, including Gβγ dimers derived from heterotrimeric G proteins, and Ras superfamily GTPases [94]. These interactions are often very weak in solution, with tight complex formation only occurring on membrane surfaces. The reason for increased affinity on membranes is still not entirely understood, with it likely being through a combination of both increased local concentration and unique conformational changes that occur on membranes, which may promote protein binding. HDX-MS has been a useful tool to analyze how the GTPase HRas and lipidated Gβγ recruit the p110γ and p110β catalytic isoforms [108–110] to membrane mimetics. Interpretation of these HDX-MS experiments is complicated because exchange differences can be caused by both direct protein–protein contacts and enhanced recruitment to membranes. Defining the Gβγ binding sites required careful controls where membrane binding can be promoted in the absence of Gβγ, and looking for unique decreases in exchange caused by Gβγ. However, even with these challenges, this approach identified the correct Gβγ binding site in both PI3Kγ [111] and PI3Kβ [112], based on recent computational and cryo-EM analysis.

### HDX-MS analysis of antibody-epitope interactions

An area of study where HDX-MS can be advantageous from a pharmaceutical perspective is its ability to effectively map antigen-antibody interactions. Development of antibodies and nanobodies as drugs is common in the pharmaceutical industry [113,114], with AI-enabled methods used to generate high-throughput predictions of antibody-antigen interactions. In addition, novel computational tools are allowing for the *in silico* design of antibodies/nanobodies towards specific epitopes on a target protein. However, both the prediction and design of epitope-antibody pairs suffer from a high percentage of false positives in most AI-enabled methods [115]. Therefore, experimental methods are still essential in validating both designed and predicted antibody/nanobody interfaces, with HDX-MS being invaluable in this approach. This is one of the most common applications of HDX-MS in the biopharmaceutical industry, with multiple reviews examining the opportunities and pitfalls in the use of HDX-MS to map epitope-binding sites [4,62,63,115].

**Figure 5:**
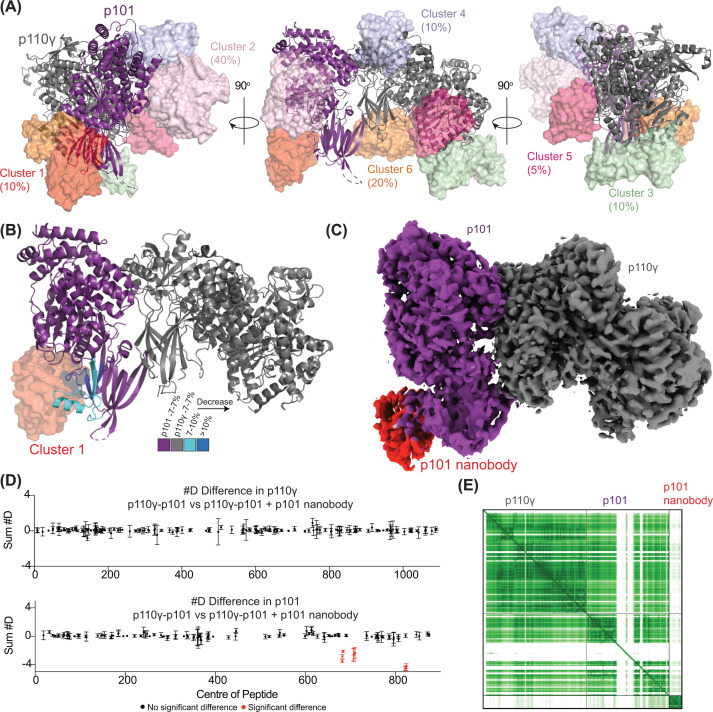
Combining AI prediction of nanobody epitopes with HDX-MS and cryo-EM (**A**) Overlay of 20 models of PI3Kγ in complex with the p101 nanobody obtained from four different seeded AlphaFold3 searches, pLDDT < 50 removed from all structures. Six unique clusters of nanobody placement are observed, with the percentage of models corresponding to each cluster annotated. Configurations where the nanobody was not shown in contact with PI3Kγ have been removed (5% of models). (**B**) Changes in HDX mapped onto an AlphaFold3 structure where the p101 nanobody epitope matched cluster 1 predictions. (**C**) Cryo-EM density map of PI3Kγ in complex with the p101 nanobody. The molecular model of the p101 nanobody bound to p101 from the electron density map was made possible by using an AlphaFold predicted structure, as the density did not permit for manual building. Due to the wide range of predicted epitopes, HDX-MS was critical to verify which predicted structures were most correct. (**D**) Sum of the #D difference in p110γ and p101 over the entire time course. Significant changes are only observed in p101, having no direct interactions with p110γ. (**E**) AlphaFold predicted alignment error plot for the prediction shown in panel (B). This figure contains data from [61] and [108].

A clear example of how HDX-MS effectively screens AI-generated structural predictions of antigen-antibody interactions is from analysis of how a p101-selective nanobody binds to the p110γ–p101 complex (heterodimeric complex referred to as PI3Kγ) (Figure 5). This complex represented a well-validated nanobody interface, with a cryo-EM map of the nanobody bound to the PI3Kγ complex [108], as well as HDX-MS data of PI3Kγ with and without the bound nanobody [61]. The original cryo-EM map did not have sufficient local resolution to model the nanobody; hence, no existing PDB model existed of this complex to bias structure prediction results. Four distinct AlphaFold3 searches of the PI3Kγ-nanobody complex are shown in Figure 5A, with this representing a total of 20 possible nanobody poses bound to PI3Kγ. The 20 models clustered into 6 distinct groups (Clusters 1–6) (Figure 5A), suggesting a possible six unique epitopes from AI validation. When comparing these predictions to HDX-MS results of PI3Kγ bound to the nanobody, only cluster 1 fits this data (Figure 5B,D). It is important to note that the cluster 1 epitope was only predicted in 10% of the resulting models, and the AlphaFold PAE plots were essentially equivalent for all predictions (Figure 5E). This highlights that it is extremely challenging to predict the correct epitope with predictive AI alone. The orientation of the nanobody in cluster 1 fits extremely well into the cryo-EM density map of the PI3Kγ-nanobody complex (Figure 5C). In this instance, the use of HDX-MS significantly streamlined the process of determining this epitope. AI has enabled an explosion in the number of structural predictions that can be produced for a spectrum of biotherapeutics, making high-to-medium throughput techniques, which can validate these predictions, sorely needed. Altogether, coupling structure prediction and HDX-MS provides a streamlined and cost-effective platform for the screening of antigens and antibodies, bridging the gap between broad-based antibody–antigen screening techniques and the rigorous structural and functional characterization of the most promising candidates.

## Protein small molecule interactions

Differential HDX-MS is a powerful approach for probing protein-small molecule interactions [2,4,116]. While most discussions of HDX-MS for ligand binding have focused on mapping binding sites, there is also a key role in defining unexpected allosteric conformational changes upon compound binding. Many of the enzymes targeted by small molecule inhibitors exist as dynamic conformational ensembles, sampling multiple states rather than a single rigid structure. Many small molecule inhibitors act by manipulating this ensemble, shifting it toward a restricted subset of conformations—a process often invisible in static structures. HDX-MS can be useful to both map direct binding sites and provide information on ligand-driven allosteric changes. The following sections highlight examples where HDX-MS has uncovered allosteric changes that were undetected by traditional structural methods.

Therapeutic targeting of proteins often involves locking the protein into a specific state with small molecules promoting distinct conformations, with a key example being ATP-competitive type I versus type II kinase inhibitors, which target either the active DFG-in or inactive DFG-out state [117,118]. However, frequently these inhibitors show very similar conformations by X-ray crystallography or cryo-EM, but show dramatic differences in protein dynamics by HDX-MS [59,60,119–121]. HDX-MS has been extensively applied to reveal ligand-driven allosteric changes not observed in static structural models. Across multiple protein classes, HDX-MS has detected changes distal to binding pockets, often at key regulatory interfaces. This demonstrates that small-molecule binding can induce previously invisible alterations in secondary structure that influence protein function by stabilizing or mimicking specific conformations within the protein’s structural ensemble.

A key example of how distinct small molecule classes can cause dramatic differences in protein conformation is provided by HDX-MS analysis of distinct classes of PI3Kγ inhibitors [60]. This study looked at seven total inhibitors, with most showing similar overall protein structures when co-crystallized with PI3Kγ, with HDX-MS sorting them into three distinct groups based on their allosteric effects. The first group produced only localized protection near the kinase hinge region, consistent with direct inhibitor binding. The second group caused protection at the active site along with increased exchange throughout the kinase domain. The third group triggered widespread structural destabilization, highlighting a significant role for allostery in PI3Kγ regulation. Intriguingly, inhibitors that caused the largest structural destabilization were some of the most isoform-selective, showing that HDX-MS analysis can be harnessed to enhance inhibitor selectivity [122]. Similarly, the serine/threonine kinase Akt has been structurally characterized bound to multiple inhibitors using the isolated kinase domain. However, these structures lack the PH domain, which forms an autoinhibitory interface with the kinase domain. HDX-MS revealed that ATP-competitive inhibitors binding to Akt’s ATP pocket drive large-scale allosteric changes, particularly at the PH-kinase domain interface [59]. ATP-competitive Akt inhibitors are known to increase activating T308 and S473 phosphorylation, with this disruption of the kinase-PH interface providing a possible molecular mechanism of action [123].

Beyond clarifying inhibitor binding sites, HDX-MS can reveal unique conformational states and regulatory surfaces that may provide additional insight into the molecular mechanism(s) of inhibition. Proteins function by transitioning between such states, many of which are transient and difficult to characterize with traditional methods. HDX-MS can detect these hidden states, showing how small molecules can stabilize, mimic, or exclude specific conformations within the ensemble. This insight is invaluable for therapeutic design: inhibitors can be engineered not only to compete with substrates but also to exploit conformational dynamics, targeting regulatory ensembles that remain cryptic in static structures.

### Using HDX-MS to define structural dynamics of disease-linked mutations

One of the most promising applications of HDX-MS is its ability to provide valuable structural insight into mutant-induced conformational changes. These effects on structural stability can often be nuanced and cause large allosteric conformational changes [102,124]. HDX-MS has been used to investigate mutations found in cancer and disease across a multitude of signaling pathways with the overarching goal of providing the molecular mechanisms underlying human disease [125–128]. It has proven to be powerful in studying gain-of-function mutations associated with cancer or diseased states. These mutations often mimic activation states by disrupting inhibitory interactions or promoting catalytically competent states. The ability of HDX-MS to help identify how mutations promote specific activating conformations can be exploited to develop oncogene-selective biological inhibitors as next-generation therapeutics.

**Figure 6:**
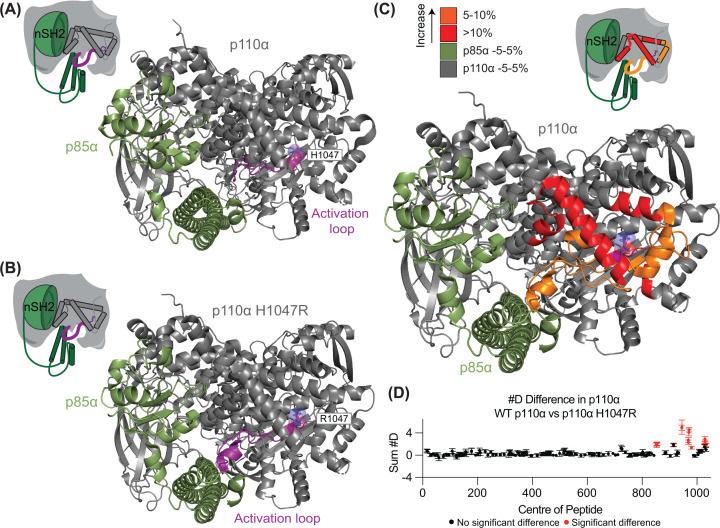
HDX-MS can detect mutant-induced changes in structural dynamics (**A**) Structure of wild type p110α in complex with p85α (PDB 7MYN) [131]. (**B**) Structure of mutant H1047R p110α in complex with p85α (PDB 8GUB) [132]. The activation loop is shown in purple in both panels (A) and (B). (**C**) Differences in HDX between WT PI3Kα and the H1047R mutant mapped onto the mutant structure (PDB 8GUB) [132]. (**D**) Sum of the #D difference in p110α over the entire time course when comparing the WT and H1047 mutant constructs, showing increased exchange within the kinase domain and regulatory motif, and activation loop. This figure contains data from [102].

The following section highlights how HDX-MS has been used in this way, providing insight into potential therapeutic sites of oncogenic mutants [102,129] and has provided mechanistic insight into mutant-dependent variations in inhibitor sensitivity and therefore contributed to the optimization of how current therapeutics are implemented in the clinic [130]. This section will demonstrate how the use of HDX-MS provided unique structural information largely invisible by other techniques to characterize which state of protein activation an oncogenic mutation mimics. The second most frequently mutated gene in all human cancers is *PIK3CA*, which encodes the catalytic p110α subunit of PI3Kα, with one of the most prominent oncogenic mutations being H1047R. HDX-MS analysis of the oncogenic H1047R PI3Kα mutant revealed extensive destabilization of functionally important secondary structure across its kinase domain (activation loop and regulatory arch), indicated by increased exchange in these regions relative to the wild type (Figure 6). Despite the availability of a crystal structure for the H1047R mutant, this destabilization was not apparent when compared with WT PI3Kα, which showed only a conformational change in the activation loop (Figure 6A,B). These observed destabilizations likely enhance catalytic activity by stabilizing an open conformation that favors membrane recruitment and promotes a catalytically competent state. In this case, HDX-MS provided a level of structural insight unattainable through crystallographic analysis alone, underscoring its utility in resolving dynamic features of oncogenic H1047R PI3Kα. HDX-MS thus provides a means to uncover mutant-specific conformations that can be, and have been, exploited for H1047R mutant-specific development.

## Conclusion

HDX-MS is firmly established across academia and industry as a powerful, versatile tool for probing protein structure and dynamics. Continued advances in instrumentation and automation have streamlined the methodological workflow, with recent advances in automated data analysis suggesting we may soon reach its full potential in biochemical and structural studies. This review emphasizes how HDX-MS is a powerful complementary tool to X-ray, cryo-EM, and AI-enabled modeling. By far the biggest change in modern structural biology is the rapid rise of AI-based protein modeling, which has transformed our ability to predict conformational landscapes and protein interfaces. HDX-MS provides an experimental approach that directly reports on dynamics and protein interfaces, offering critical validation for computational models. As AI methods continue to accelerate, complementary biophysical experimental approaches will remain essential, and HDX-MS is uniquely positioned to play an increasingly central role in future structure-guided discovery.

## Open Access

Open access for this article was enabled by the participation of the University of British Columbia in an all-inclusive *Read & Publish* agreement with Portland Press and the Biochemical Society.

## Abbreviations

ACBD3: acyl-CoA binding protein 3
ARMH3: armadillo-like helical domain containing 3
AI: artificial intelligence
cryo-EM: cryo-electron microscopy
HDX: Hydrogen–deuterium exchange
HDX-MS: Hydrogen–deuterium exchange mass spectrometry
IDRs: intrinsically disordered regions
MS: mass spectrometry
PAE: predicted aligned error
PPIs: protein–protein interactions
TGN: trans-Golgi network
TTC7B: Tetratricopeptide repeat protein 7B
PI4KA: phosphatidylinositol 4-kinase alpha
PI4KB: phosphatidylinositol 4-kinase beta
PI3Ks: phosphoinositide 3-kinases
PI3Kδ: phosphoinositide 3-kinase delta
PI3Kγ: phosphoinositide 3-kinase gamma
PI3Kα: phosphoinositide 3-kinase alpha
SH2: Src homology 2
H/D: hydrogen-deuterium
Gβγ: G protein βγ subunit
PDB: protein data bank
